# Loose Tooth, a Way to Save It: A Case Report

**DOI:** 10.5152/TJAR.2022.1120

**Published:** 2022-04-01

**Authors:** Kamran Mottaghi, Farhad Safari, Sara Nashibi, Masoud Nashibi

**Affiliations:** 1 Department of Anaesthesiology, Shahid Beheshti University of Medical Sciences, Loghamn Hakim Hospital, Tehran, Iran; 2 Shahid Beheshti University of Medical Sciences Faculty of Dentistry, Tehran, Iran

**Keywords:** Laryngoscopy, loose tooth, mouth guard, nasal splint, tracheal intubation

## Abstract

A loose tooth is a great concern for anaesthesiologists either as a potential foreign body or a bleeding source. A 48-year-old male patient scheduled to undergo a lumbar discectomy had a loose maxillary incisor; he got his tooth fixed by using a thermoplastic external nasal splint. Different approaches such as modification in laryngoscopy or removal of loose teeth have been proposed, but fixing and keeping it in place is not a usual practice which was successfully applied for our patient. Loose incisors could be fixed and protected by using a thermoplastic nasal splint as a mouth guard.

**Main Points**

- A loose tooth may not only be a potential foreign body but also an impediment to laryngoscopy and tracheal intubation.
- Fixing a loose tooth in the operating room could be a challenge to anaesthesiologists.
- A thermoplastic nasal splint could be used effectively as a mouth guard to keep a loose tooth safe and in place for a couple of hours in the operating theater.

## Introduction

Loose teeth are a real concern for anaesthesiologists since they could be a potential foreign body and may be aspirated, as a source of bleeding or even a legal claim. When dislodged, the tooth must be sought, located, and removed as soon as possible. Different approaches are proposed to fix a loose tooth or to avoid its dislodgement.^1,2^ Hereby we report an urgent case, whose loose tooth was fixed with an innovative approach.

## Case Presentation

**Figure 1.**
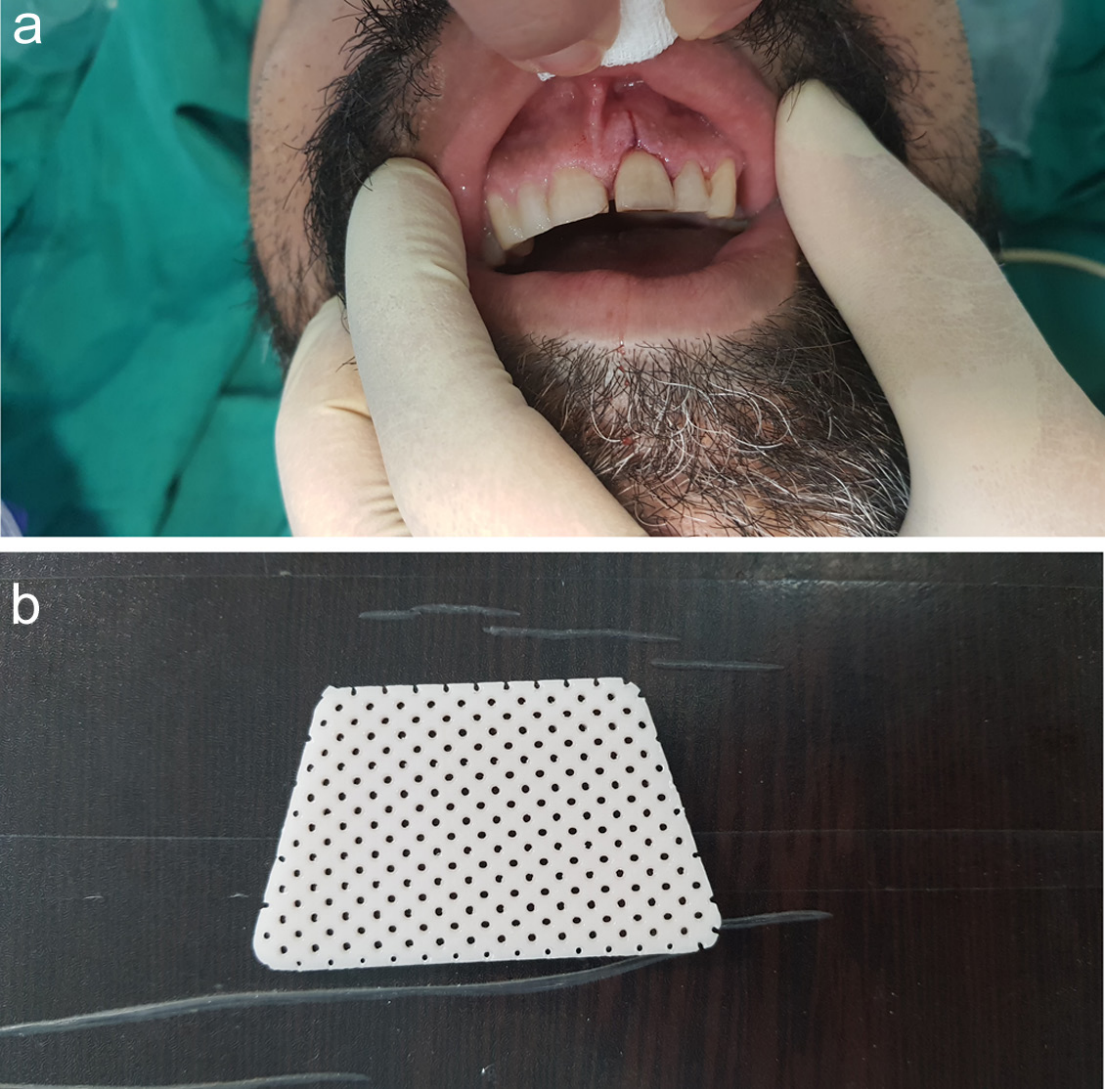
(A) Loose central left maxillary incisor. (B) Thermoplastic external nasal splint.

**Figure 2.**
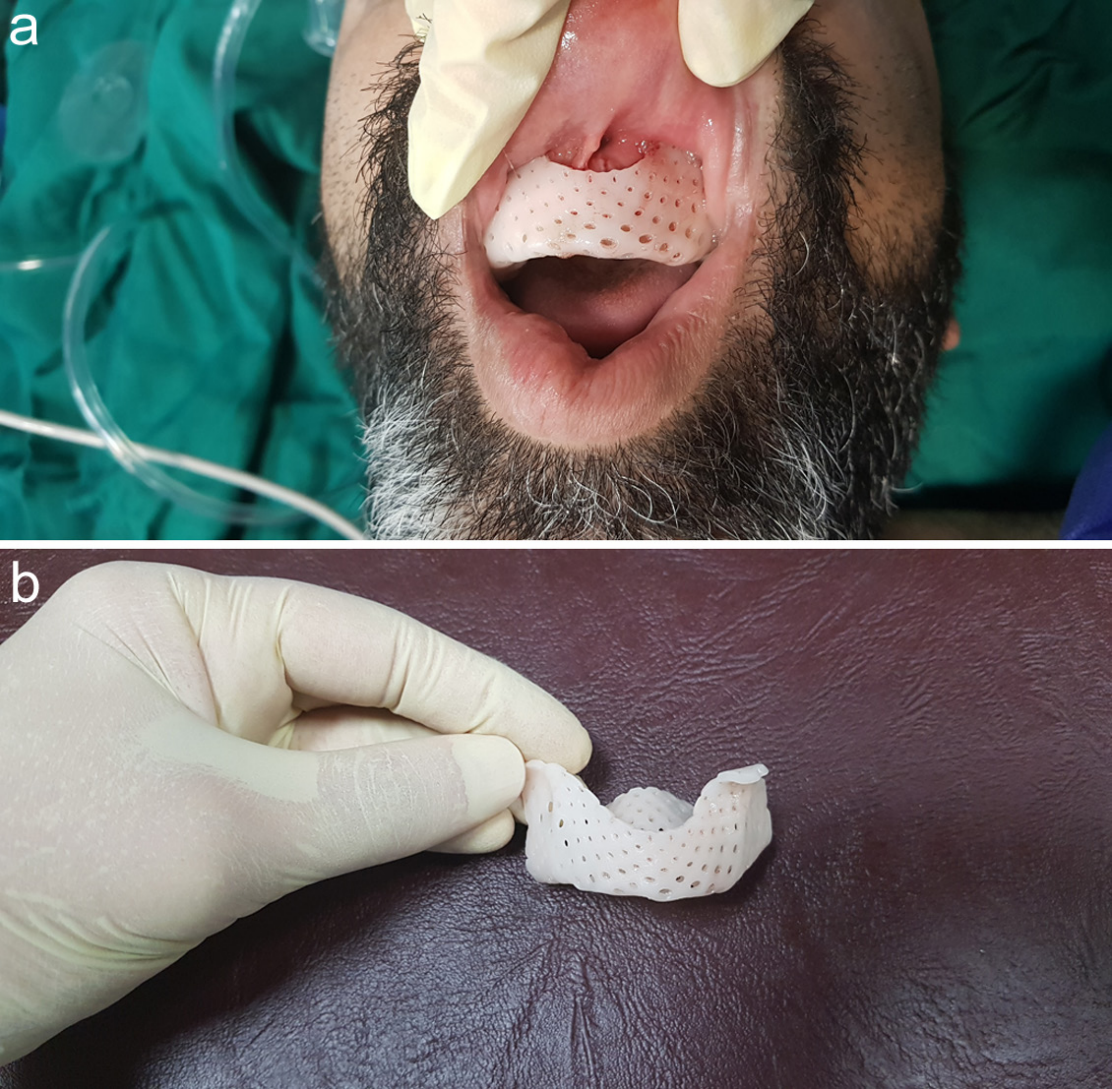
(A) Thermoplastic external nasal splint molded as mouth guard. (B) Removed molded mouth guard.

A 48-year-old otherwise healthy man was admitted for urgent lumbar discectomy secondary to lumbar disc herniation which was represented with paresthesia and decreased force of lower extremities. The patient had a loose left maxillary incisor due to recent minor trauma during the prayers ritual (Figure 1A). This loose tooth was prone to direct damage and dislodgement during direct or video laryngoscopy. We used a thermoplastic external nasal splint (Asanmed Tehran-Iran) (Figure 1B) to cover and fix this tooth. After softening in warm water, it was left to cool down to 37°C and applied over the maxillary incisors and canines. Then, put cold tap water-soaked gauze in order to mold and harden it like a rigid mouth guard (Figure 2A). Then, we started with premedication by using midazolam 0.03 mg kg^-1^ and fentanyl 3 µg kg^-1^ followed by induction with propofol 2 mg kg^-1^ and atracurium 0.5 mg kg^-1^. Finally, the intubation was done using a video laryngoscope without giving any harm to that tooth. Anaesthesia was maintained by using propofol 200 µg kg^-1^ min^-1^ to keep the cerebral state index (CSI) between 40 and 60. The mouth guard was left in place till the end of surgery while the patient was in prone position for about one hour. Residual muscle relaxation was reversed by injecting neostigmine 0.05 mg kg^-1^ and atropine 0.02 mg kg^-1^. After emergence, the patient was under observation in post-anaesthesia care unit. Before patient’s discharge to ward, the mouth guard was gently removed from his tongue (Figure 2B); physical examination showed no bleeding or damage to the loose incisor tooth. Informed written consent was obtained from the patient to publish data.

## Discussion

Using thermoplastic splints to cover a loose tooth, although simple, economic, and affordable, is a competent way to keep it in place when urgent procedures may not provide enough time for an elective dentistry consultation.

Thirty-nine percent of anaesthesia-related claims are due to dental damage; the most common dental damage involved the fracture to incisors.^3^ Tracheal intubation, per se, could be a potential danger to the teeth, especially during difficult laryngoscopies. Therefore, anaesthesiologists try to avoid teeth damage by using different approaches such as putting a rolled gauze over the teeth or covering them with mouth guards,^4^ or using paraglossal technique.^5^ Loose teeth are even more vulnerable to direct damage and dislodgment and as a potential foreign body, source of bleeding or a legal case is a concern to anaesthesiologists.^5,6^ Different approaches, such as fixing with a thread,^2^ removing of a loose tooth,^1^ or modified laryngoscopy and intubation efforts,^1^ have been proposed to decrease the likelihood of dislodgment and losing loose teeth.

Thermoplastic mouth guard has been presented before to prevent veneer or enamel damage during difficult laryngoscopy.^4^ General tendency of the removal of loose teeth may be wise among children since the primary teeth will be lost in near future; however, in adult patients, it should not be the first choice. Some authorities propose modified approaches to laryngoscopy in order to avoid direct damage while others just mark it with a thread to make it easy to find it after dislodgment. The threat is not limited to the time of laryngoscopy as the tooth could be dislodged during mouth suctioning and tracheal extubation. Since rigid oral airway may also damage the teeth, some of the anaesthesiologists prefer to use a nasal airway to avoid dental damage during mask ventilation or in the post-anaesthesia care unit.^5^Although we warned the patient about possible loss of the tooth, he was grateful to the medical team for keeping the tooth in place and sought dental care after discharge.

## Conclusion

Using a thermoplastic external nasal splint could help to fix a loose incisor in place during laryngoscopy, tracheal intubation, and throughout the surgery in prone position and make it safe to use rigid oral airway during mask ventilation.
